# DNA Methylation of *Igf2r* Promoter CpG Island 2 Governs *Cis*-Acting Inheritance and Gene Dosage in Equine Hybrids

**DOI:** 10.3390/biology14060678

**Published:** 2025-06-11

**Authors:** Xisheng Wang, Yingchao Shen, Hong Ren, Minna Yi, Gerelchimeg Bou

**Affiliations:** 1 Lin He’s Academician Workstation of New Medicine and Clinical Translation, Jining Medical University, Jining 272067, China; 2 Anchee (Shandong) Animal Nutrition Research Academy Co., Ltd., Jinan 250104, China; shenyingchao2017@163.com; 3 Vocational and Technical College, Inner Mongolia Agricultural University, Hohhot 010018, China; renhong1980@126.com; 4 Equus Research Center, Inner Mongolia Agricultural University, Hohhot 010018, China; yiminna2020@163.com (M.Y.); gerelchimeg@imau.edu.cn (G.B.); 5 College of Animal Science, Inner Mongolia Agricultural University, Hohhot 010018, China

**Keywords:** *Igf2r*, DNA methylation, equus, *cis*-acting inheritance, genomic imprinting, epigenetic regulation

## Abstract

Genomic imprinting is critical for mammalian development, but its regulation varies across species. The insulin-like growth factor 2 receptor (IGF2R), which is a maternally expressed imprinted gene critical for cell proliferation and differentiation, as well as embryonic and placental development, is classically regulated by differentially methylated regions (DMRs) and lncRNA-*Airn* in mice. However, studies on this in equus are scarce, especially in terms of mechanistic studies. In the present study, heart, liver, spleen, lung, kidney, brain, and muscle samples were obtained from horses, donkeys, and hybrids, and gene expression and imprinting state were tested to investigate the imprinting regulation of *Igf2r* in these animals. Bisulfite sequencing combined with an allele-specific expression analysis revealed a tissue-specific loss of imprinting in the mule liver and hybrid brain tissues. Strikingly, we found that the maternal-specific expression of equine *Igf2r* did not rely on the canonical DMRs or lncRNA-*Airn*. Surprisingly, DNA methylation of a specific region called CpG island 2 (CpGI2) in the *Igf2r* promoter showed *cis*-acting inheritance, meaning that the DNA methylation patterns of the parental alleles are retained in hybrid tissues. Notably, the DNA methylation of CpGI2 correlated negatively with *Igf2r* expression in the spleen (R^2^ = 0.8797, *p* = 6.46 × 10^−6^), lung (R^2^ = 0.8569, *p* = 1.57 × 10^−5^), and kidney (R^2^ = 0.8650, *p* = 3.85 × 10^−6^). Our findings suggest that imprinting may work differently in other species. This study provides a framework for understanding imprinting diversity in hybrids and shows that equine hybrids can be used to study how epigenetic inheritance works.

## Simple Summary

The *Igf2r* expression pattern in offspring is influenced by the parents and is important for organism growth. In this study, we examined the *Igf2r* gene in horses, donkeys, and their hybrids. We found that the parent-specific expression of *Igf2r* was lost in equine brain and mule liver tissues, whereas it was retained in most other tissues. Surprisingly, this was not related to canonical differentially methylated regions (DMRs). However, DNA methylation patterns in CpG island 2 (CpGI2) in the promoter of *Igf2r* could be transmitted from parents to offspring, thereby determining its expression activity, even in hybrids. This helps us to improve hybrid breeding strategies and address livestock growth issues.

## 1. Introduction

In mammals, genomic imprinting is an epigenetic phenomenon, leading to the silencing of one of the parental alleles, thereby resulting in the monoallelic expression of numerous genes [1,2]. Imprinted genes have been demonstrated to play critical roles in the regulation of growth, metabolism, and brain development. Dysregulation of these genes has been linked to the development of developmental disorders and diseases [3,4]. DNA methylation imprints are erased in primordial germ cells and re-established differentially during gametogenesis. These regions are known as germline differentially methylated regions (gDMRs) and can resist genome-wide demethylation during preimplantation development. They are also referred to as imprinted control regions (ICRs). Subsequently, ICRs determine the expression of a non-coding gene and multiple coding gene single alleles in a cluster [2]. Therefore, to resolve the regulatory mechanism of imprinted gene expression, studying its DNA methylation pattern is indispensable. Notably, DNA methylation patterns can be inherited, independent of canonical ICRs [5]. Such *cis*-acting methylation directly modulates gene expression by inhibiting transcription factor binding or recruiting methyl-binding proteins, thereby suppressing promoter activity [6,7].

One classic maternal transmission imprint gene, the insulin-like growth factor 2 receptor (IGF2R) gene, mainly encodes a receptor responsible for insulin-like growth factor 2 (IGF2) degradation, and disruption of its maternal transcription results in over-growth and perinatal lethality [8]. Therefore, the *Igf2-Igf2r* axis plays a direct role in the regulation of cell proliferation, fetal growth, and placental development [9,10]. In addition, *Igf2r* has been implicated as a tumor suppressor gene in several human tumors, including breast cancer, hepatocellular carcinoma, and choriocarcinoma [11]. In mice, *Igf2r* imprinting is governed by two differentially methylated regions (DMR1 in the promoter and DMR2 in intron 2) and the long non-coding RNA-*Airn*, which recruits chromatin modifiers to silence the paternal allele [12,13,14]. Interestingly, *Igf2r* is biallelically expressed in humans, although the allelic-differentially methylated region in intron 2 and the non-coding RNA-*Airn* are still present [15,16].

Although *Igf2r* imprinting diverged during human and mouse evolution, equine *Igf2r* exhibits maternal-specific expression, and its regulation remains unexplored [17,18]. Furthermore, the offspring (mules and hinnies) of interspecies hybrids between horses (*Equus caballus*) and donkeys (*Equus asinus*) can offer sufficient single-nucleotide variations (SNVs) [10,19], and DNA methylation levels remain unchanged in hybrids [20] but lack identifiable *Airn* homologs in horses [21]. This system allows us to dissect imprinting mechanisms independent of conserved lncRNAs.

In our study, we found that *Igf2r* imprints were lost in the liver of mules, except in the brain tissue of mules and hinnies. Furthermore, there was no DMR, except for three CpG islands (CpGIs) in the equine *Igf2r* promoter, and intronic 2 DMRs were present in tissues, including in the liver in mules and in the brain in mules and hinnies. Given the role of *cis*-inherited DNA methylation in gene regulation [22], we hypothesize that equine *Igf2r* imprinting is independent of *Airn* and its expression is driven by *cis*-acting methylation in CpGI2 in the promoter region. To examine our hypothesis, we performed pyrosequencing, bisulfite sequencing, and absolute quantification in horses, donkeys, and their hybrids. We aimed to determine (1) whether intronic 2 DMRs regulate the imprinted expression of *Igf2r*; (2) whether promoter DNA methylation is inherited in *cis* in equids; and (3) whether the *Igf2r* expression level is dependent on its DNA methylation of CpGI2 in the promoter region. Our findings provide a framework for understanding epigenetic diversity across species.

## 2. Materials and Methods

### 2.1. Sample Collection and Preparation

All experiments and methods involving animals in this study were approved and authorized by the Animal Care and Use Committee of Jining Medical University, and all procedures were conducted in accordance with the guidelines and regulations of Jining Medical University. Tissues (muscle, liver, brain, testis, sperm, heart, spleen, lung, and kidney) were collected from well-documented adult horses (*Equus caballus*, *n* = 3), donkeys (*Equus asinus*, *n* = 3), mules (horse ♀ × donkey ♂, *n* = 3), and hinnies (donkey ♀ × horse ♂, *n* = 3) (mean age = 4 ± 0.5 years), according to ethical guidelines. All samples were immediately snap-frozen in liquid nitrogen and stored at −80 °C until processing.

### 2.2. Genomic DNA and RNA Extraction

The grinding of the tissue samples (muscle, liver, brain, testis, heart, spleen, lung, and kidney) with liquid nitrogen increased the quality and quantity of DNA and mRNA extracted from these samples. Genomic DNA was isolated from ground samples and sperm using a TaKaRa MiniBEST Universal Genomic DNA Extraction Kit (TaKaRa Bio, Dalian, China, #9765), according to the manufacturer’s protocol. DNA purity and concentration were quantified using a Take 3 plate reader (BioTek Epoch, Santa Clara, CA, USA; absorbance ratios: 260/280 > 1.8, 260/230 > 2.0). Total RNA was extracted from the heart, liver, spleen, lung, kidney, muscle, and brain tissues using TRIzol reagent (Invitrogen, Carlsbad, CA, USA, #15596026CN), followed by DNase I treatment to eliminate genomic DNA contamination. Then, 1 µg of RNA was reverse-transcribed to cDNA using a High-Capacity cDNA Reverse Transcription Kit (Applied Biosystems, Carlsbad, CA, USA, #4368814).

### 2.3. Single-Nucleotide Variant (SNV) Identification

#### 2.3.1. SNV Analysis

**Figure 1:**
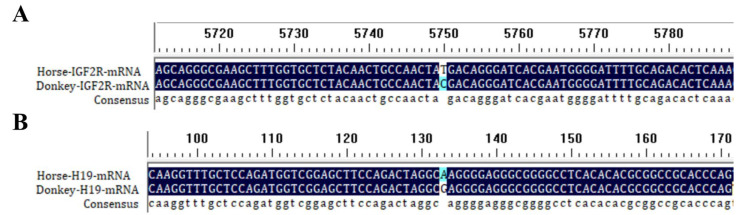
DNAMAN alignment of *Igf2r* (**A**) and *H19* (**B**) at horse and donkey mRNA SNV sites.

Reference mRNA and DNA sequences of *Igf2r* (for DNA: NCBI Accession NC_091714 for horse and NW_014638170 for donkey; for mRNA: NCBI Accession XM_005608119.4 for horse and XM_044760750.2 for donkey) and reference mRNA of *H19* (NCBI Accession: NR_027326.2 for horse; NCBI Accession: XM_014830185.1 for donkey) were aligned using BioEdit 7.0.9 to identify interspecies SNVs. Figure 1A,B show the SNVs from a comparison of *Igf2r* and *H19* mRNA sequences between horses and donkeys.

#### 2.3.2. Experimental SNV Validation

For DNA-derived SNVs, PCR amplification was performed on genomic DNA using Green Taq Mix (Vazyme, Nanjing, China, #P131), with primers flanking the SNV regions of *Igf2r*. The cycling conditions were as follows: 95 °C for 5 min; 35 cycles of 95 °C for 30 s, 60 °C for 30 s, 72 °C for 45 s; and a final extension at 72 °C for 10 min. For cDNA-derived SNVs, PCR was conducted using TB Green Premix Ex Taq (TaKaRa, Dalian, China, #RR820Q) under identical conditions. Amplicons were Sanger-sequenced (Sangon Biotech, Shanghai, China), and chromatograms were analyzed in BioEdit ver.7.0.9.0 to confirm SNVs.

### 2.4. Allele-Specific Expression (ASE) Quantification

The allele-specific expression of *Igf2r* and *H19* was quantified using pyrosequencing on a PyroMark Q48 Autoprep system (QIAGEN, Hilden, Germany) [17]. Primers (for *Igf2r*: forward: 5′-GACGCTTTGGTGCTCTAGAACTGC-3′, reverse: 5′-CCTGCCGATACCAGCATCTTCATC-3′, sequencing primer: 5′-TGCTCTACAACTGCCAA-3′, for *H19*: forward: 5′-ACAGCGAGAAGGACAATGGAATG-3′, reverse: 5′-GAGTATGCAAGAAAACTGCCGAGTG-3′, sequencing primer: 5′-CGGAGCTTCCAGACTAGG-3′ were designed with PyroMark Assay Design Software 2.0 (QIAGEN, Hilden, Germany). PCR amplification was performed in 20 µL reactions with a PyroMark PCR Kit (QIAGEN, Hilden, Germany, #978703) and the following program: 95 °C for 15 min, 45 cycles of 94 °C for 30 s, 60 °C for 30 s, and 72 °C for 30 s. Pyrosequencing data were analyzed with PyroMark Q48 Advanced CpG Reagents (QIAGEN, Hilden, Germany, #974002) using the Allele Quantification (AQ) mode. Maternal-to-paternal expression ratios were normalized to SNV-defined allelic inputs.

### 2.5. DNA Methylation Analysis

#### 2.5.1. CpG Island Prediction and Bisulfite Conversion

CpG islands in the *Igf2r* promoter and intron 2 were predicted using MethPrimer (http://www.urogene.org/methprimer, accessed on 5 March 2024). Genomic DNA (500 ng) was bisulfite-converted using an EZ DNA Methylation-Gold Kit (Zymo Research, Irvine, CA, USA, #D5006); the amount of input DNA was 200–500 ng. Conversion efficiency (non-methylated C residues are converted to U, and methylated cytosines are protected) > 99% was confirmed using the control DNA included in the kit.

#### 2.5.2. Bisulfite PCR and Cloning

Bisulfite-treated DNA (2 µL) was amplified with region-specific primers (Table 1) using ZymoTaq DNA Polymerase (Zymo Research, Irvine, CA, USA, #E2002). PCR products were cloned into the pMD™19-T vector (TaKaRa, Dalian, China, #6013), and 10–15 clones per sample were sequenced (Sangon Biotech, Shanghai, China). DNA methylation levels were quantified using QUMA (http://quma.cdb.riken.jp/, accessed on 15 March 2025). Parental allele origins were determined using an SNV analysis.

### 2.6. Absolute Quantification of Igf2r in Tissues

For the absolute quantification of reactions, a CFX96™ Real-Time System (Bio-Rad, Hercules, CA, USA) with a TB Green Premix Ex Taq II Kit (TaKaRa, Dalian, China, #RR820) was used. The *Igf2r* primer sequence was as follows: forward PCR primer: 5’-GACGCTTTGGTGCTCTAGAACTGC-3’; reverse PCR primer: 5’-CCTGCCGATACCAGCATCTTCATC-3’. At first, *Igf2r* gene fragments were cloned using the pMD™19-T vector. Further, plasmids were extracted with a TlANprep Mini Plasmid Kit (TIANGEN, Beijing, China, #DP103), and concentrations were determined using a biospectrometer (Eppendorf, Hamburg, Germany). The mass concentration of the plasmid containing the *Igf2r* target fragment was converted to a molecular copy number concentration using the following formula: Copies/mL = (6.02 × 10^23^ copies/mol) × (concentration (g)/mL)/(molecular weight (g)/mol). The quantified plasmids were subjected to 10-fold serial dilution to obtain standards in the range of 10^9^ to 10^1^, and a standard curve was plotted. An absolute real-time quantitative analysis was performed on various tissue samples, together with standard samples. The threshold cycle (Ct) value of the test sample was compared with that of the standard sample to determine the expression levels in different tissues.

### 2.7. Statistical Analysis

Data are expressed as mean ± SD. DNA methylation levels and ASE ratios were compared using a one-way ANOVA in GraphPad Prism 8.0.2. Regression analyses between DNA methylation and expression were performed using linear regression. Significance thresholds were set to *p* < 0.05.

## 3. Results

### 3.1. Igf2r Is Maternally Allele-Specifically Expressed in Adult Tissues of Mule and Hinny, Except for in the Brain of Mule and Hinny and the Liver of Mule

**Figure 2:**
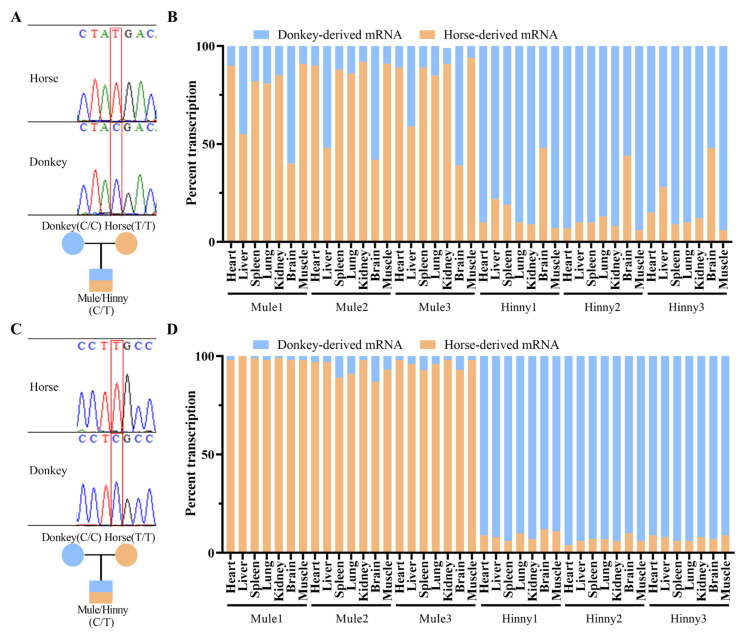
Allele-specific expression analysis of equine *Igf2r* and *H19* in hybrids. (**A**) SNV validation of *Igf2r*. (**B**) Tissue-specific ASE ratios of *Igf2r*. (**C**) SNV validation of *H19*. (**D**) Tissue-specific ASE ratios of *H19*.

Research has shown that *Igf2r* is only expressed as a biallelic gene in brain tissue, while in other tissues, it is expressed specifically as a maternal allele [23]. In our research, quantitative allele-specific pyrosequencing revealed that *Igf2r* showed strong maternal allele-specific expression in the heart, spleen, lung, kidney, and muscle tissues of both mules and hinnies (Figure 2A,B). However, imprints were lost in the liver tissue of mules, except in the brain tissue of mules and hinnies (Figure 2B). In contrast, the imprinted gene *H19* maintained stable maternal expression across all tissues (Figure 2C,D), confirming tissue-specific rather than global imprinting erosion in hybrids.

### 3.2. There Is No DMR in the Equine Promoter Region of Igf2r

**Figure 3:**
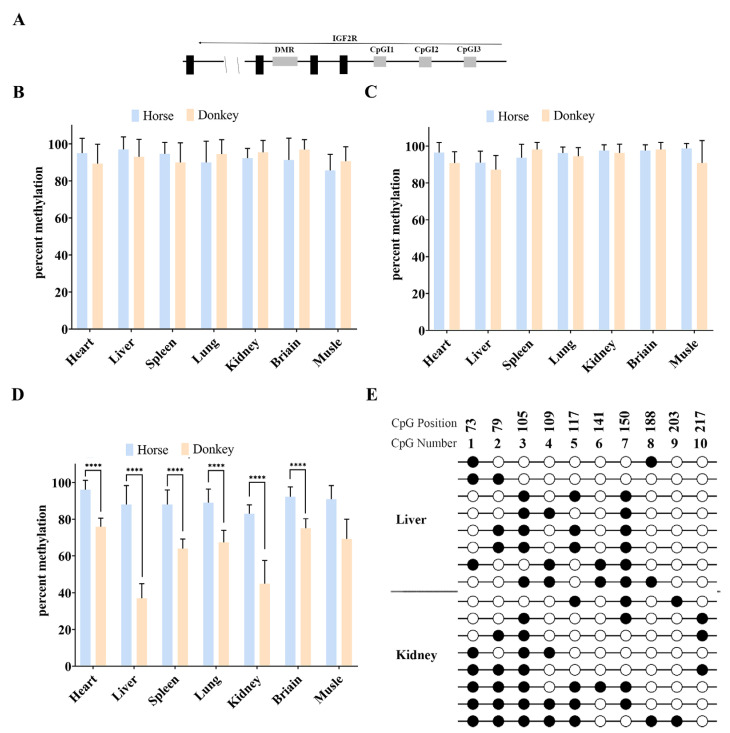
DNA methylation analysis of *Igf2r* promoter regions. (**A**) Schematic of equine *Igf2r* locus and SNV sites (black triangle), with 12 CpGs in CpGI1, 10 CpGs in CpGI2, and 16 CpGs in CpGI3 (white box). SNVs between horse and donkey are as follows: CpGI1: A (horse)/G (donkey); CpGI2: G (horse)/C (donkey); and CpGI3: A (horse)/G (donkey). (**B**) Percent methylation of CpGI1 in the *Igf2r* promoter. (**C**) Percent methylation of CpGI3 in the *Igf2r* promoter. (**D**) Percent methylation of CpGI2 in the *Igf2r* promoter. Effective readings ≥ 10 (****: *p* < 5.48 × 10^−5^). (**E**) Bisulfite sequencing of CpGI2 in the *Igf2r* promoter with donkey tissues (liver and kidney). Effective readings = 8.

Bisulfite and Sanger sequencing were performed on DNA from the horse and donkey tissues. The results showed that CpG islands (CpGIs) 1 and 3 in the *Igf2r* promoter region were all hypermethylated (66.7–100% methylation) without differential DNA methylation in the parent of origin (Figure 3A–C). Species-specific hypermethylation differences were observed in the CpGI2 region, but hypermethylation was maintained in the horse tissues, and tissue-specific hypermethylation differences were observed in the donkey tissues, demonstrating that DMRs were not present in the horse promoter region (Figure 3D). Further assays in the donkey liver and kidney tissues showed that the donkey promoter region was also free of DMRs (Figure 3E).

**Figure 4:**
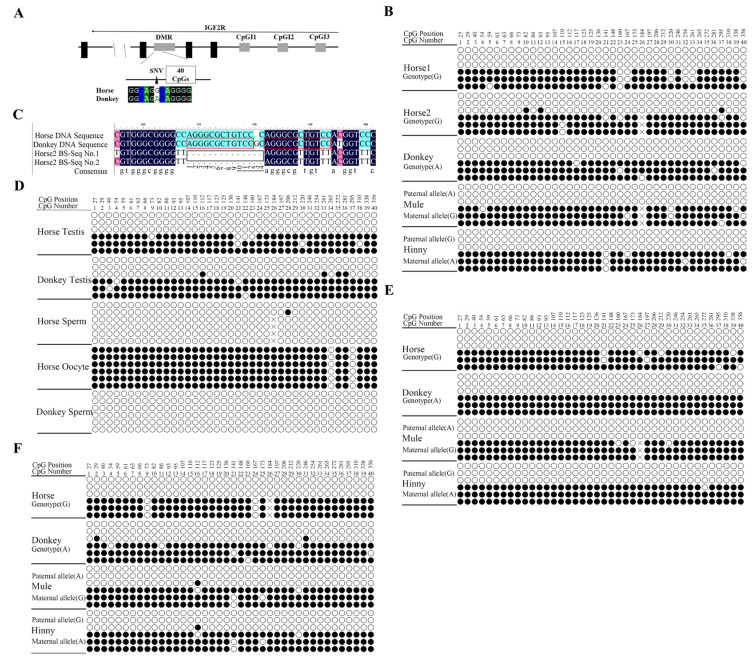
DNA methylation analysis of *Igf2r* promoter and intronic regions. (**A**) Schematic of equine *Igf2r* locus, including transcriptional direction (arrow), transcription unit (black box), DMR, and CpG islands (gray box). Bisulfite mutagenesis and sequencing are indicated below, including 1 SNV site (black triangle) and 40 CpGs (white box). SNVs between horse and donkey are as follows: G (horse)/A (donkey). (**B**) Maternal allele-specific methylation in intron 2 of *Igf2r* in equine hearts; × represents the deletion of a 14 bp repetitive sequence fragment (AGGGCGCTGTCCGC). Each row of circles represents an individual strand sequenced. Black circles represent methylated cytosines, white circles represent unmethylated cytosine. (**C**) Comparative results of bisulfite sequencing (BS-Seq) of the horse2 intron 2 DMR region: 14 bp deletions are shown in black boxes, and 12 bp repeat sequences are shown in red boxes. (**D**) Conservation of the intronic DMR in germ cells (sperm and oocytes). (**E**) Maternal allele-specific methylation in intron 2 of *Igf2r* in the liver. (**F**) Maternal allele-specific methylation in intron 2 of *Igf2r* in the brain. Effective readings ≥ 6.

Studies on the heart tissues of horses, donkeys, mules, and hinnies revealed a maternal allele-specific DMR in the intron 2 region of *Igf2r* in equines (Figure 4A,B), and this DMR remained unaltered by a 14 bp deletion in the horse (Figure 4B,C). Subsequent studies on testicular tissues and germ cells (sperm hypermethylation: 0.42 ± 0.1%; oocytes: 95 ± 0.1%) revealed that the DMR in the *Igf2r* intron 2 region was a gametic DMR (Figure 4D) and persisted in the liver and brain tissue (Figure 4E,F). A collective analysis of these results indicated the presence of a maternal allele-specific DMR in the intron 2 region of *Igf2r* in equids, which does not regulate the imprinted expression of *Igf2r*.

### 3.3. Cis-Acting Sequences Govern DNA Methylation Inheritance in Equines

**Figure 5:**
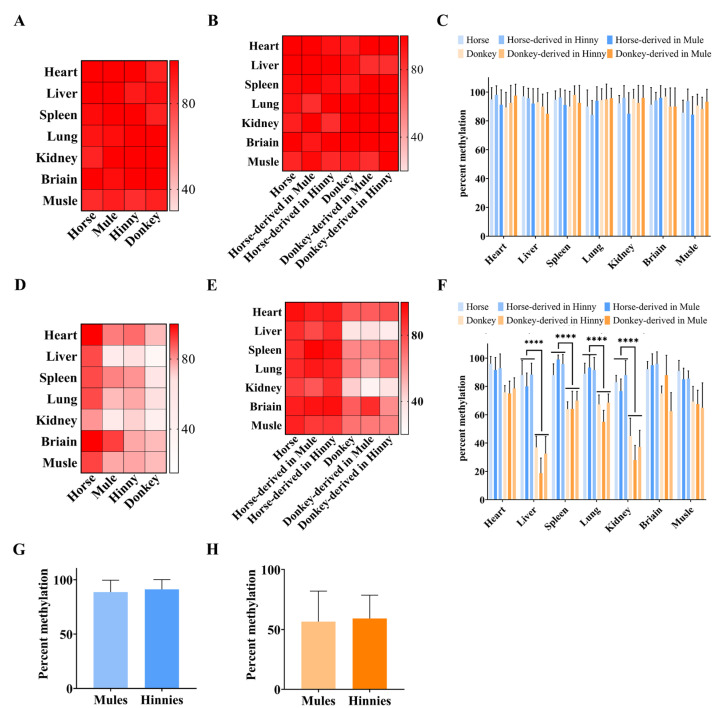
DNA methylation *cis*-acting inheritance of *Igf2r* in promoter CpGI2 in equines. (**A**) DNA methylation rates of seven equine tissues (heart, liver, spleen, lung, kidney, brain, and muscle) in CpGI1. (**B**) DNA methylation rates in CpGI1, and DNA methylation in hybrid tissue traced back to parents using SNV information. (**C**) Bar chart: DNA methylation rates in CpGI1. (**D**) DNA methylation rates in CpGI2, tending towards the result of horse > hybrids > donkey. (**E**) DNA methylation rates in CpGI2. (**F**) Bar chart: DNA methylation rates in CpGI2, (****: *p* < 6.46 × 10^−7^). (**G**) DNA methylation rates of horse-derived alleles of *Igf2r* in CpGI2 in hybrid tissues. (**H**) DNA methylation rates of donkey-derived alleles of *Igf2r* in CpGI2 in hybrid tissues. *n* = 3.

DNA methylation can be divided into two types: imprinted DNA methylation and nonimprinted DNA methylation. The imprinted DNA methylation of the DMR of the *Igf2r* intron 2 region is mainly determined by *cis*-acting sequences. However, we found the same pattern in the *Igf2r* promoter region in equine. Bisulfite and Sanger sequencing showed that the DNA methylation differences between horses and donkeys were not significant in CpGI1 and CpGI3 (Figure 3B,C), while they were significant (*p* < 5.48 × 10^−5^) in CpGI2 (Figure 3D). We then investigated the DNA methylation patterns of CpGI1 and CpGI2. The sequencing results showed that CpGI1 DNA methylation exhibited a high methylation state between horses, donkeys, mules, and hinnies (Figure 5A). However, CpGI2 showed species-specific DNA methylation, tending towards the result of horse > hybrids > donkey (Figure 5D). Further research with SNV information showed that high DNA methylation was maintained uniformly across species in CpGI1 (Figure 3A and Figure 5B), with a DNA methylation rate of 71–100% (Figure 5C). In contrast, hybrid tissues retained parental DNA methylation patterns at CpGI2 (Figure 3A and Figure 5E), with horse-derived alleles maintaining hypermethylation (88.66 ± 10.88% in mules and 91.16 ± 8.89% in hinnies) (Figure 5F,G) and donkey-derived alleles showing hypomethylation (56.63 ± 25.48% in mules and 59.2 ± 19.5% in hinnies) (Figure 5F,H). These results underscore that CpGI2 DNA methylation is dictated by species-specific *cis*-regulatory elements.

### 3.4. Promoter CpGI2 DNA Methylation Drives Igf2r Expression Level in a Tissue-Dependent Manner

**Figure 6:**
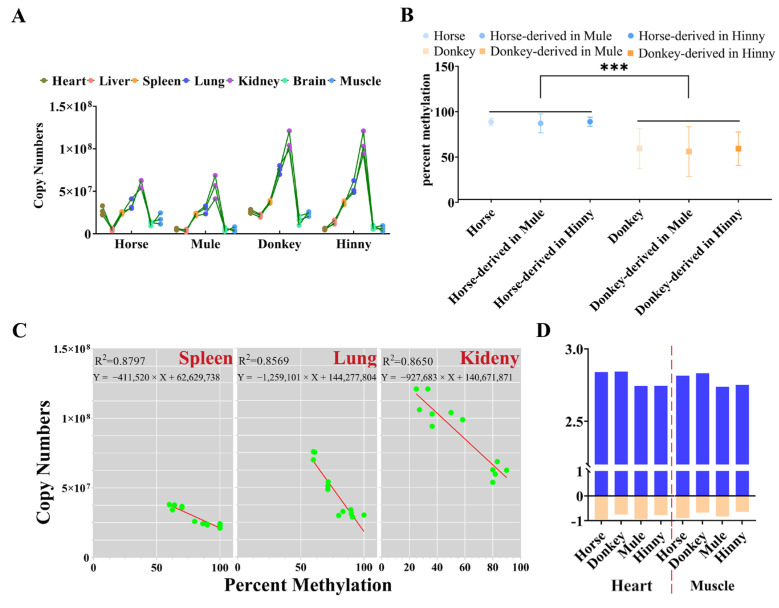
Tissue-dependent correlation between CpGI2 DNA methylation and *Igf2r* expression. (**A**) *Igf2r* expression levels in mules and hinnies mirror their respective maternal species. (**B**) CpGI2 DNA methylation states align with parental origin (***: *p* = 1.82 × 10^−4^). (**C**) Linear regression between DNA methylation and expression of *Igf2r* in spleen, lung, and kidney. (**D**) Relationship between DNA methylation and *Igf2r* gene expression. Data transformation methods: copy number values were subjected to double natural log transformation (LN(LN)), while DNA methylation levels were normalized to a range of 0–1, followed by × −1 adjustment.

DNA methylation has been identified as the principal regulator of gene function, playing a pivotal role in developmental processes and over the course of an individual’s lifetime [24,25,26]. A real-time PCR analysis revealed that the expression levels of *Igf2r* in mules were analogous to those observed in horses, while hinnies exhibited a profile consistent with that of donkeys (Figure 6A). Furthermore, the results showed that the DNA methylation of CpGI2 of *Igf2r* in the promoter region in mules or hinnies was consistent with the origin being from horses or donkeys (Figure 6B). Linear regression analyses revealed a strong negative correlation between CpGI2 DNA methylation and *Igf2r* expression in the spleen (R^2^ = 0.8797, *p* = 6.46 × 10^−6^), lung (R^2^ = 0.8569, *p* = 1.57 × 10^−5^), and kidney (R^2^ = 0.8650, *p* = 1.10 × 10^−5^) (Figure 6C). In the heart and muscle tissues, DNA methylation and expression exhibited weaker correlations in combined linear regression analyses across all species. Nevertheless, when subjected to separate analyses, negative correlations between DNA methylation and expression were observed in horses and donkeys, as well as in mules and hinnies (Figure 6D). All results suggest that the DNA methylation in the promoter region and expression of *Igf2r* in horses and donkeys can be passed down from generation to generation to mules and hinnies, which also indicates that the DNA methylation in the promoter region of *Igf2r* can regulate its expression.

## 4. Discussion

The maternal-specific expression of *Igf2r* and its epigenetic regulation in rodents are well described, whereas in our study, we identified a unique mode of regulation in equine hybrids. Here, we conducted a comparative study of the imprinting and DNA methylation status of *Igf2r* in the equine genome, focusing on allele-specific expression, promoter CpG island methylation, and the intron 2 DMR. By integrating bisulfite sequencing and allele-specific expression assays, we demonstrated that maternal *Igf2r* imprinting is present in the majority of somatic tissues of horses and donkeys despite the lack of promoter DMRs and *Airn* homologues, which is in stark contrast to typical mouse models, suggesting that equine mammals diverged during evolution.

While the maternal allele-specific DMR in intron 2 (DMR2) is conserved in equines, its functional divergence from murine models raises questions about its potential role in gene dosage regulation. Our study identified a maternally inherited DMR in intron 2 of equine *Igf2r* (DMR2), which is conserved across mice, humans, and equines. While this DMR persists in somatic and germline tissues, its methylation status shows no correlation with *Igf2r* expression levels. In mice, the intronic DMR2 interacts with lncRNA-*Airn* to silence the paternal allele by recruiting chromatin modifiers [27]. Intriguingly, we demonstrated that CpGI2 methylation, not canonical DMR2, drives *Igf2r* dosage in equines. This suggests that equines have evolved a regulatory axis independent of conserved lncRNA-dependent silencing, potentially driven by metabolic or placental adaptations [28,29,30].

The absence of *Igf2r* imprinting in mule liver and brain tissues raises critical questions about tissue-specific epigenetic stability. In mice, brain-specific imprint relaxation has been attributed to neuron-specific histone modifications and *Airn* deficiency [23]. Similarly, we observed that equine brain tissue exhibits biallelic *Igf2r* expression, likely due to chromatin histone modifications during neurogenesis. However, the sole absence of imprints in the mule liver—but not in the hinny liver—suggests interference due to hybridization [31]. In the present study, we found that the most substantial disparities in the parental DNA methylation of the *Igf2r* promoter CpGI2 locus are present in mule liver tissue. It is evident that DNA methylation plays a pivotal role in various biological processes, including gene expression, histone modifications, transcription factor binding, and chromatin accessibility [32,33,34]. Consequently, it can be hypothesized that this could be a contributing factor to the observed absence of *Igf2r* imprinting in mule liver tissue. Future research should analyze specific histone modifications, chromatin accessibility, and transcription factors in this tissue to test this hypothesis.

While mouse *Igf2r* has a differentially methylated region (DMR) in its promoter to regulate parent-of-origin expression [12], unlike mice, equine *Igf2r* lacks promoter DMRs, suggesting lineage-specific adaptation [35]. Interestingly, all three species—mouse, horse, and human—share a conserved DMR within intron 2. However, humans show a critical divergence: despite the persistence of this intronic DMR, *Igf2r* lost its imprinted status and switched to biallelic expression [35]. This juxtaposition highlights a dual evolutionary trajectory: the conservation of regulatory architecture and species-specific adaptations, reflecting both functional constraint and lineage-specific innovation in genomic imprinting mechanisms.

Specific DNA sequences contribute to the *cis* inheritance of epigenetic modifications and gene expression [36,37]. Our data indicate that the CpGI2 DNA methylation pattern is inherited in-*cis,* and even in hybrid cells, and parental alleles can maintain a species-specific DNA methylation status. This is in stark contrast to the imprinting DMR, which is typically reset during germ cell development [38]. The stability of DNA methylation *cis*-inheritance suggests that DNA methylation is closely linked to SNVs and DNA motifs [5,39,40]. This mechanism may represent a primitive but evolutionarily conserved strategy to maintain parental epigenetic memory. It is worth noting that the strong negative correlation between CpGI2 DNA methylation and *Igf2r* expression in the spleen, lung, and kidney highlights its regulatory role, while the weaker correlation in the muscle and heart may reflect tissue-specific differences in enhancers or co-regulatory factors [41]. Future studies mapping enhancer landscapes across tissues will clarify how *cis*-regulatory elements interact with CpGI2 methylation to fine-tune dosage [42].

While our hybrid model provides unique insights, several limitations warrant attention. First, the absence of *Airn* in equines does not exclude the involvement of other lncRNAs. Second, the functional causality between CpGI2 DNA methylation and *Igf2r* expression remains correlative. CRISPR-mediated DNA methylation editing or allele-specific knockout experiments are needed to provide direct evidence [43]. Comparative analyses of DNA methylation and gene expression patterns across species may yield more robust conclusions when validated within the same tissue type of a single species, minimizing the confounding variables inherent in inter-species comparisons. Despite these limitations, our work establishes equine hybrids as a powerful model for studying *cis*-acting epigenetic inheritance.

## 5. Conclusions

In this study, we investigated the mechanisms by which DNA methylation in the promoter and intron 2 of *Igf2r* regulates its expression in equids. Through comparative epigenetic analyses of horses, donkeys, and their hybrids (mules and hinnies), we obtained the following results: (1) A conserved maternal allele-specific DMR in *Igf2r* intron 2 was identified in horses, but it was not associated with imprinted expression patterns. (2) There was no DMR in the *Igf2r* promoter region, but the DNA methylation of CpG island 2 (CpGI2) showed *cis*-acting inheritance, and the parental methylation pattern was retained in hybrids. (3) CpGI2 methylation was strongly negatively correlated with *Igf2r* expression in spleen, lung, and kidney tissues (R^2^ > 0.85, *p* < 0.001), suggesting a regulatory role. These findings broaden our comprehension of epigenetic diversity across mammals and underscore the utility of equine inbreeding models for investigating genomic imprinting mechanisms. Future research combining multi-omics strategies and functional assays will further clarify how sequence-specific DNA methylation influences developmental pathways and evolutionary adaptations in hybrids and beyond.

## Figures and Tables

**Table 1:** Primers used to detect methylation levels.

Region	Primer Sequence (5′-3′ Orientation)	Annealing Temperature (°C)	Product Size (bp)
CpGI1	F: GGGGGAGGGTTTTTAAGG	57	321
	R: AATAATAACAAATTTCAAAACTAACC		
CpGI2	F: AGATAATTTGTGTAAGAGGGAATATTTT	60	259
	R: CCAATCTACCCCTAATCTATCCTTCCTA		
CpGI3	F: GTGGTTTAGGGGTGGAGATTA	54	362
	R: TATTCTCCAAACTACCCCCTTCC		
Intron 2	F: GGTTATTTTGTTTTTGGGTTTGTATAG	58	341
	R: TCTCTTCTTAAAAATCAAATCACAT		

## Data Availability

The original contributions presented in this study are included in the article. Further inquiries can be directed to the corresponding author.
